# Progress and challenges of the bioartificial pancreas

**DOI:** 10.1186/s40580-016-0088-4

**Published:** 2016-11-01

**Authors:** Patrick T. J. Hwang, Dishant K. Shah, Jacob A. Garcia, Chae Yun Bae, Dong-Jin Lim, Ryan C. Huiszoon, Grant C. Alexander, Ho-Wook Jun

**Affiliations:** 1 Department of Biomedical Engineering, University of Alabama at Birmingham, Birmingham, AL 35294 USA; 2 806 Shelby, 1825 University Boulevard, Birmingham, AL USA; 3 Department of Otolaryngology, University of Alabama at Birmingham, Boshell, 1720 2nd Avenue South, Birmingham, AL 35294 USA

**Keywords:** Bioartificial pancreas, Macroencapsulation, Microencapsulation, Islet surface modification, Microfluidic, Micropatterning

## Abstract

Pancreatic islet transplantation has been validated as a treatment for type 1 diabetes since it maintains consistent and sustained type 1 diabetes reversal. However, one of the major challenges in pancreatic islet transplantation is the body’s natural immune response to the implanted islets. Immunosuppressive drug treatment is the most popular immunomodulatory approach for islet graft survival. However, administration of immunosuppressive drugs gives rise to negative side effects, and long-term effects are not clearly understood. A bioartificial pancreas is a therapeutic approach to enable pancreatic islet transplantation without or with minimal immune suppression. The bioartificial pancreas encapsulates the pancreatic islets in a semi-permeable environment which protects islets from the body’s immune responses, while allowing the permeation of insulin, oxygen, nutrients, and waste. Many groups have developed various types of the bioartificial pancreas and tested their efficacy in animal models. However, the clinical application of the bioartificial pancreas still requires further investigation. In this review, we discuss several types of bioartificial pancreases and address their advantages and limitations. We also discuss recent advances in bioartificial pancreas applications with microfluidic or micropatterning technology.

## Background

Diabetes mellitus type 1, or type 1 diabetes, is a widespread disease where individuals are unable to produce the insulin necessary to process blood glucose because of an autoimmune response which destroys the body’s insulin-producing beta cells [1]. Insulin therapy has been used to treat type 1 diabetes patients since the discovery of insulin in 1922. However, daily insulin treatments are not able to precisely and continuously meet the demands of the uncontrolled variations in stress, food intake, and physical activities [2]. As an alternative treatment, pancreatic islet transplantation has been attempted to maintain consistent and sustained type 1 diabetes reversal. Successful pancreatic islet transplantation does not require rigorous blood glucose monitoring and also prevents the progression of diabetic complications [3]. However, one of the primary challenges facing pancreatic islet transplantation is the body’s natural immune response towards the foreign islets. Implantation of islets from donors can give rise to rapid immune response when exposed to the recipient immune system [4, 5]. Moreover, the traditional approach in which immunosuppressive drugs are administered during and after islet transplantation has been known to cause many side effects, such as oral ulcers, peripheral edema, anemia, weight loss, and episodic diarrhea [4, 6]. Thus, approaches for islet transplantation therapy without use of immunosuppressive drugs are desired. To address this issue, many groups have attempted to encapsulate islets within bioartificial pancreases [7–9]. A bioartificial pancreas encapsulates the pancreatic islets in a semi-permeable environment and prevents islet exposure to the body’s immune responses while allowing the permeation of insulin, oxygen, nutrients, and waste products [9]. Prevention of contact between the transplanted islets and immunocompetent cells can reduce cell-mediate immunity. Several types of bioartificial pancreases have been investigated which utilize macro- and microencapsulation [9]. In addition, engineering of the islet surface is another immunoisolation approach in which the surface of islets is modified to form an immune barrier [7]. Although various kinds of bioartificial pancreases have been developed, clinical outcomes are still not clear. In this review, we describe bioartificial pancreases and islet surface modification approaches, and address their advantages and limitations as immunoisolation strategies. We also discuss recent advances in bioartificial pancreas applications with microfluidic or micropatterning technology.

## Review

### Macroencapsulation of pancreatic islets

**Fig. 1:**
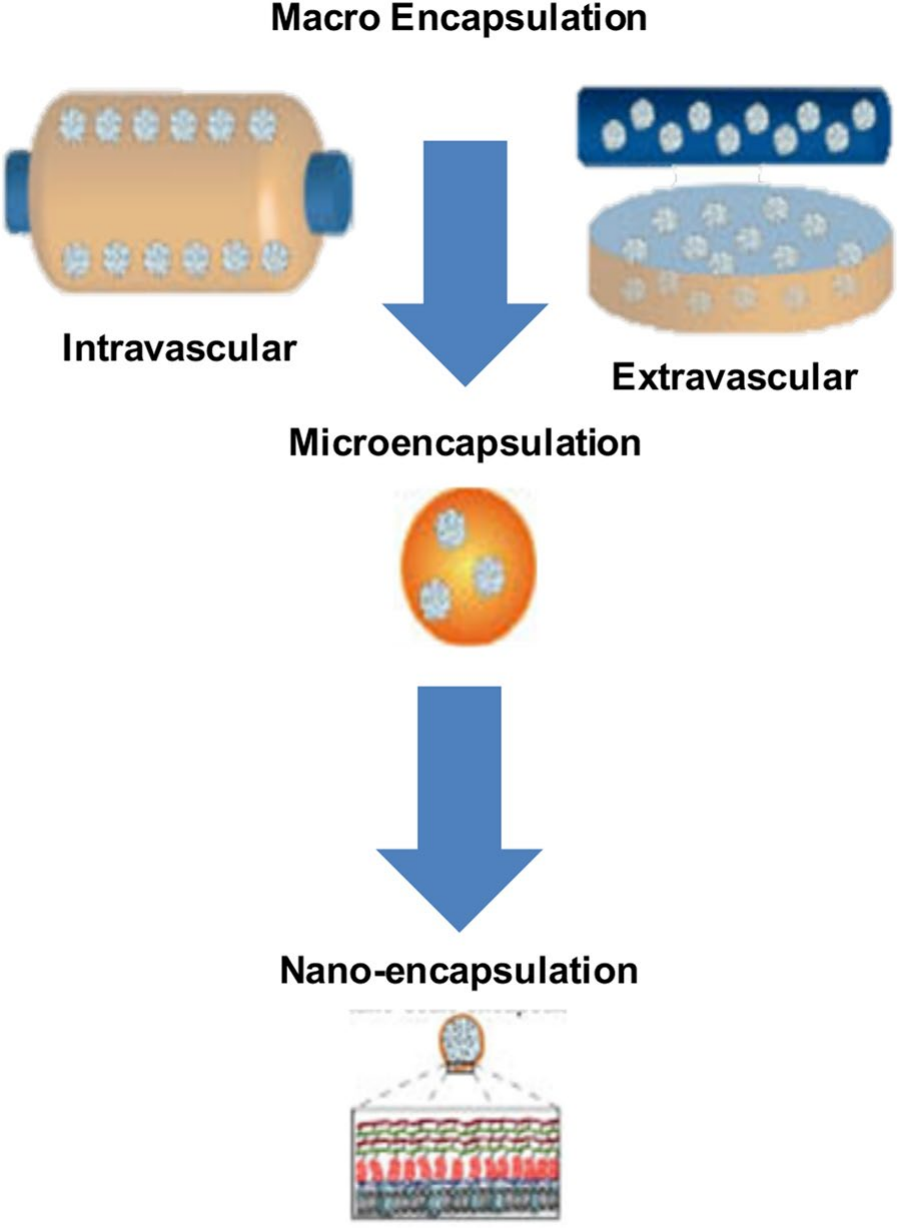
Summary of encapsulation from macro- to nanoscale. Reused with permission from [10]

Immunoisolation of pancreatic islets is generally divided into three different methods of encapsulation: macro-, micro-, and nanoencapsulation (islet surface modification at nano-scale) (Fig. 1) [10]. Macroencapsulation is the transplantation of a large number of islets within an implantable device (either extravascular or intravascular) [11]. Transplantation sites for the macrocapsule vary based on the application. Extravascular macrocapsules are implanted intraperitoneally or subcutaneously, while intravascular macrocapsules are implanted around a vessel and exposed to systemic blood flow [11]. In this review, the evolution of macroencapsulation will be discussed based on extravascular and intravascular implantation.

#### Extravascular application of macroencapsulation device

##### Diffusion chamber

**Fig. 2:**
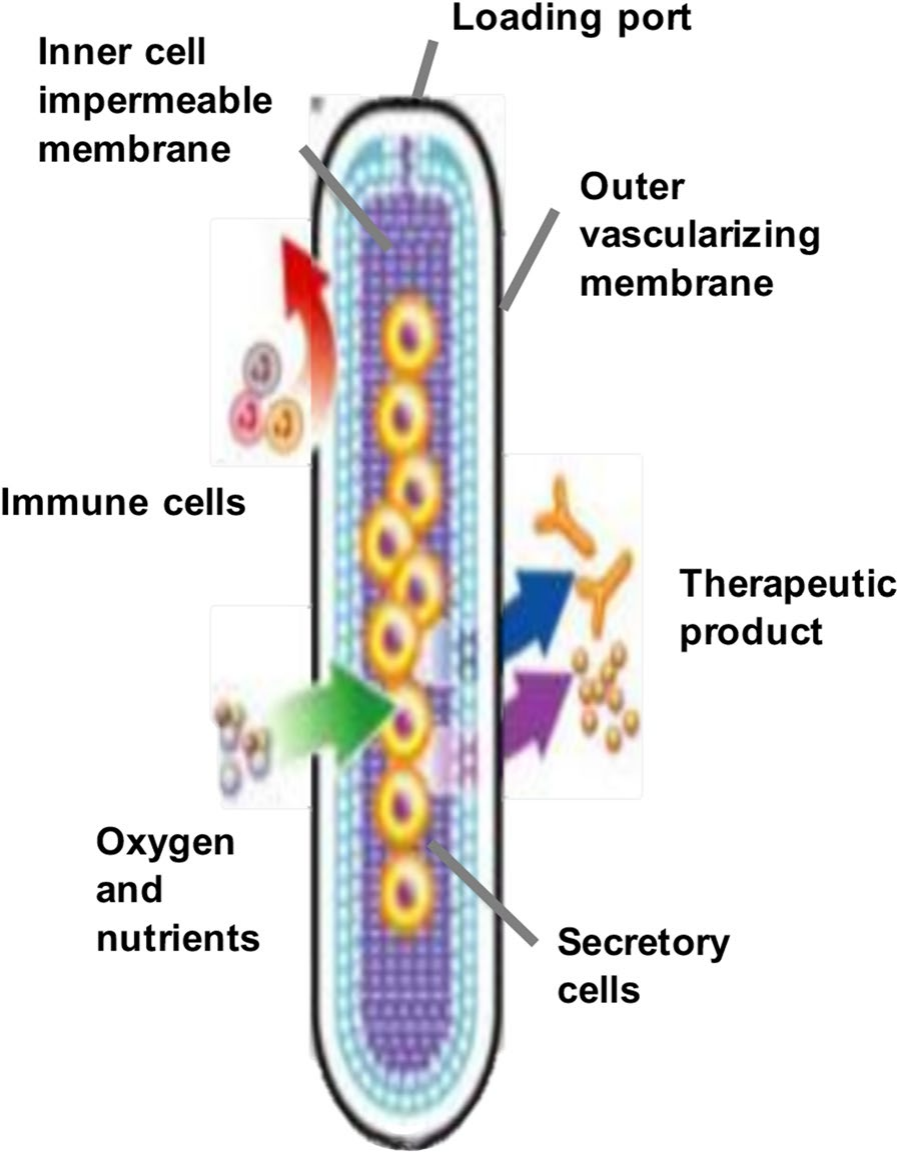
TheraCyte diffusion chamber. Reused with permission from [11]

Extravascular approaches of macroencapsulation began with the diffusion chamber. The diffusion chamber provided an artificial barrier against larger lymphocytes and macrophages, while allowing passage of smaller nutrients, gases, and insulin. One of the well-studied diffusion chamber designs is the TheraCyte bio-artificial pancreas (Fig. 2) [11]. The TheraCyte system is made from polytetrafluoroethylene (PTFE) and is composed of a planar, bilaminar membranous pouch. The inner, cell impermeable, immunoisolation membrane has a pore size of 0.4 µm; the outer membrane, utilized for tissue engraftment, has a pore size of 5 µm [11, 12]. The TheraCyte diffusion chamber is implanted at a subcutaneous site where insulin can diffuse into the blood through the membrane [12], while the islets are protected from the host’s immune system [12]. The subcutaneous placement allows for ease of surgical access and retrieval if necessary. Additionally, the islets exist as clusters within the macrocapsule diffusion chamber (as in the native pancreas), which allows for cell-to-cell communication and has shown to be beneficial for insulin production from pancreatic beta cells.

A study was done in which rat islets were encapsulated in the TheraCyte device and implanted within non-obese diabetic (NOD) mice [13]. The implanted islets survived and showed response to glucose levels, maintaining sufficient insulin secretion to normalize blood glucose levels in hyperglycemic diabetic rats at least 50 days [13]. Through this result, the encapsulated rat islets within the TheraCyte device were shown to maintain their function. However, there is concern for clumping of the islets, which could potentially reduce oxygen and nutrient flow to the interior of the islets [11]. The interior cells can become necrotic, which results in a significant deficiency in insulin production within the diffusion chamber [12]. Since islets generally need to be located within 150–200 µm of a blood vessel, this is a common weakness of the extravascular implantation device [14]. This lack of vascularization can cause increased diffusion times for insulin, potentially leading to insulin inhibition within the islets due to a build-up of insulin in the diffusion chamber [11]. Thus, reduction of clumping and enhanced vascularization within the device are areas of ongoing research.

Another study was conducted using a diffusion chamber with a bi-laminar encapsulation device implanted within streptozotocin (STZ)-induced diabetic rats [15]. These rats all received roughly eight million MIN6 cells (pancreatic beta cell-line) in a xenograft macroencapsulation diffusion chamber. Those rats that received the macrocapsule diffusion chamber showed recovery to normal glycemia up to 30 weeks post transplantation, as well as associated weight gain [15].

##### Layered alginate sheets with islets

**Fig. 3:**
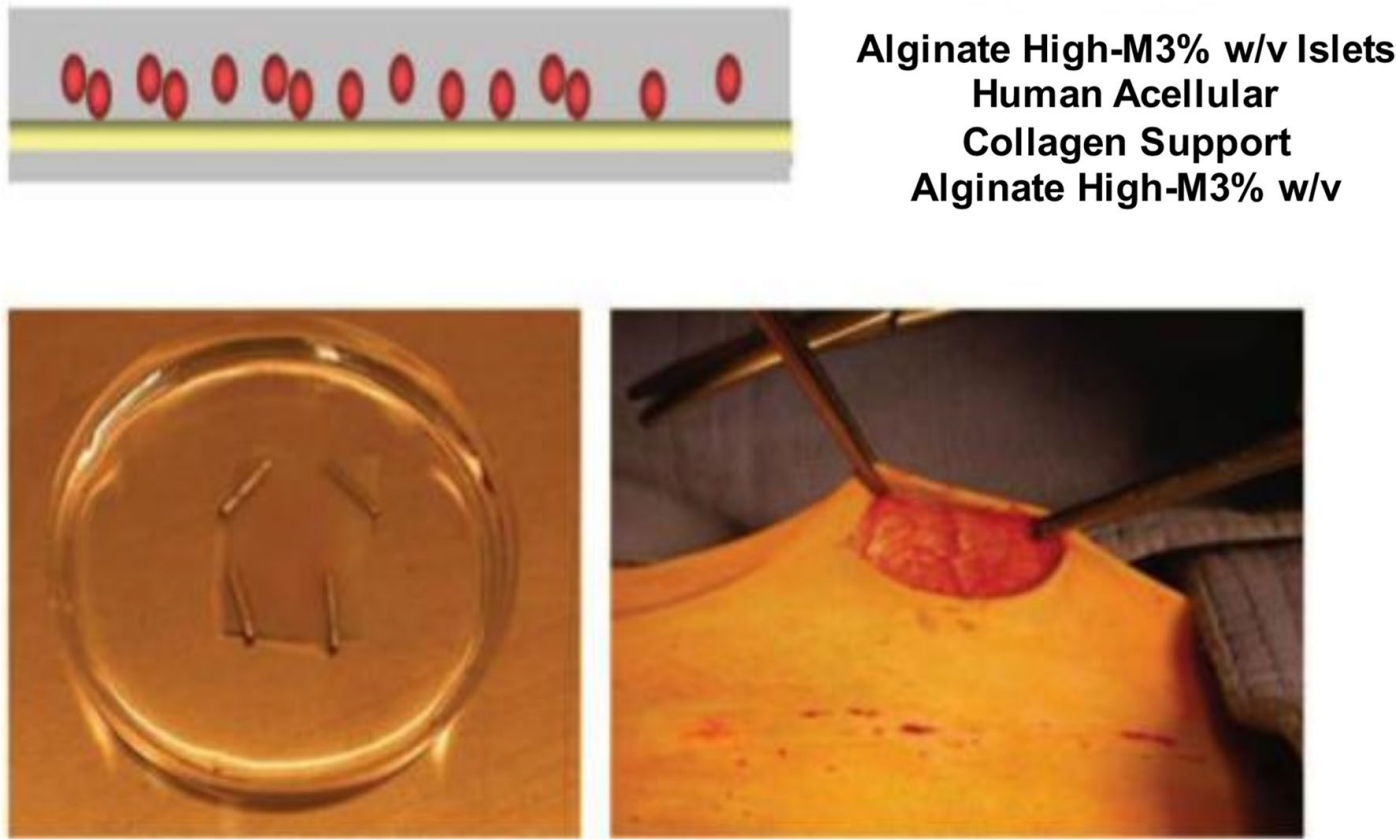
Schematic representation of alginate macrocapsule containing a monolayer of islets (*top*). Alginate macrocapsule with islets (*bottom*, *left*); Implantation of alginate macrocapsule into subcutaneous tissue of monkey (*bottom*, *right*). Reused with permission from [15]

A possible remedy to the hypoxic environment seen in clumping of islet cells is immobilization of islets in layered alginate sheets. Alginate is a hydrogel consisting of anionic polysaccharides extracted from seaweed, which confers excellent biocompatibility [16]. This layered technique inhibits clumping while allowing adequate numbers of islets to be implanted. The disadvantage of using alginate as a hydrogel is the variability associated with its pore size due to its sourcing from organic seaweed [11]. A recent study was conducted using an alginate layering device with porcine islets implanted into STZ-induced diabetic monkeys (Fig. 3) [16]. The five monkeys received three to five monolayer alginate devices, and they showed reversal of hyperglycemia for up to 28 weeks. Histological evaluation of the monolayer cellular device showed little graft fibrosis or alginate degradation. However, CD3 stained lymphocytes and CD9 stained macrophages were found within the macrocapsule along with increased levels of anti-porcine antibodies shortly after implantation [16].

#### Vascular application of macroencapsulation device

##### Coiled hollow fiber tube

**Fig. 4:**
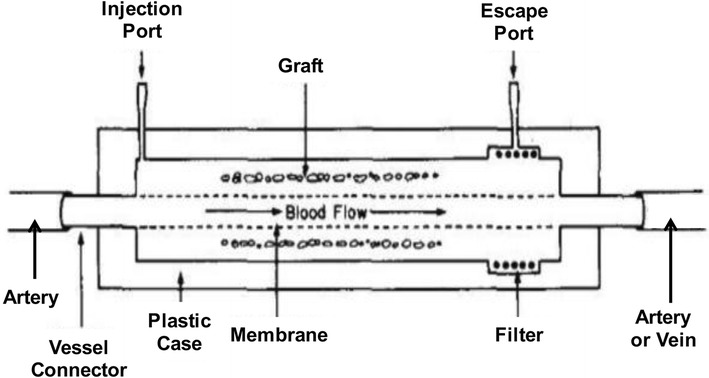
Intravascular hollow fiber tube diffusion chamber. Reused with permission from [19]

**Fig. 5:**
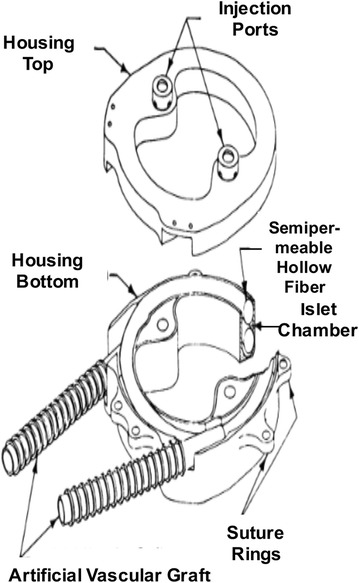
Intravascular coiled hollow fiber tube within acrylic disc shaped housing. Reused with permission from [18]

The vascular approach to macroencapsulation offers a whole new set of advantages and disadvantages. With the incorporation of the vascular system into the macrocapsule device, nutrients, oxygen, and insulin become more readily available, resulting in better blood glucose control [11]. However there is greater risk of thrombosis and fibroblast growth on the membrane with the closer proximity to blood flow [17]. In addition, the surgical procedure is more invasive and poses greater risks [11]. A common intravascular application device is a hollow fiber tube contained in a housing connected to the host vasculature (Fig. 4) [18, 19]. The islets are placed inside the device and on the interior of the semipermeable membrane. Glucose, oxygen, nutrients, and insulin can pass freely across the membrane. The immune barrier is retained as lymphocytes and immunoglobulins cannot cross the membrane, protecting islet viability. The longevity of this device is increased by the incorporation of two syringe ports in the acrylic disk-shaped housing, which allows the addition of islets after surgical implantation of the device (Fig. 5) [18].

In one study, fifteen diabetic pancreatomized dogs received a hollow fiber tube device with endogenous canine islets (as shown in Fig. 5); twelve dogs had an initial return of fasting blood glucose levels to normoglycemia, and seven of those showed long-term normal fasting blood glucose levels (100–284 days) [18]. The eventual failure of the hybrid artificial pancreas device was attributed to loss of islet viability. Diabetic canine recipients of multiple (two) hollow fiber tube devices with endogenous islets were also evaluated via intravenous glucose tolerance tests (IVGTT). The dogs that received two devices showed even greater IVGTT results than the single device implantation group [18]. In another study, implantation of the vascularized coiled hollow fiber tube device with allogeneic islets resulted in limited success; bovine and porcine islets implanted in pancreatomized dogs showed low islet viability and function (3–16 days) [18].

Another study was done involving nineteen human diabetic patients: a nylon-macrocapsule hollow tube device with fetal rabbit islets was implanted in the forearm cubital vein [20]. These implanted devices resulted in positive reversal of diabetes in fourteen of the patients for two years post-implantation, showing a 60–65 % decrease in exogenous insulin needed and a complete disappearance of hypo- or hyperglycemia related comas [20]. There was some delay in neoangiogenesis which resulted in initial islet cell loss due to insufficient vascularization of the membrane for two weeks post-transplantation. Another possible concern for this intravascular device is the increased chance of thrombosis across the membrane, leading to decreased diffusion rates [18]. Thus, the use of anticoagulants is unadvisable in patients with this type of implant device due to increased bleeding risk.

The intravascular diffusion chamber has undergone revision and evolution resulting in another approach to engraftment: the ultra-filtration system, which eliminates any prolonged diffusion times for insulin, nutrients, and oxygen. This greater filtration, however, still has similar issues with clogging and thrombosis as the membrane, and can also be affected by buildup of proteins [19].

#### Current macroencapsulation application

##### Synthetic hydrogel

Current applications of macrocapsules are on the cutting edge of biomaterial technology. As opposed to the organic alginate hydrogel, synthetic hydrogels and thermoplastics have become more widely utilized for immunoisolation within macroencapsulation devices, due to their controllable properties as a membranous barrier against the immune system [21, 22].

Synthetic hydrogels function as a water infused network of hydrophilic polymers or copolymers, which act as a membranous barrier for a macrocapsule device [23]. Due to their viscoelasticity and high H_2_O content, they mirror the attributes of natural biological tissues and usually elicit only a limited or no immune inflammatory response [23]. The hydrogel’s structural integrity is built and dependent upon crosslinking between polymer chains. This crosslinking results in adjustable pore size, which is a desirable characteristic for selective immunoisolation [23].

Additional applications can be seen in thermoplastic membranes. Thermoplastic membranes are comprised of linear water insoluble chains, which can be configured into various forms through cyclic heating and cooling processes [11]. Thermoplastics provide greater chemical and mechanical stability compared to hydrogels [11]. However, hydrogels remain the most common material used for cell encapsulation because of the advantage in biocompatibility.

##### Inorganic membranes (Al/Al_2_O_3_, Si, Ti/TiO_2_)

Inorganic membranes for islet encapsulation have become another ongoing field of research. Three inorganic materials currently have desirable properties for the creation of immunoisolating membranes: silicon (Si), aluminum/aluminum oxide (Al/Al_2_O_3_) and titanium/titanium oxide (Ti/TiO_2_) [24]. The use of these inorganic membranes as extravascular macrocapsule devices confers several advantages over their polymer alternatives, such as a higher tolerance pore size distribution and more effective diffusion due to a decrease in membrane thickness [25]. The use of an inorganic membrane composed of Al_2_O_3_ to encapsulate islets is also advantageous because it offers a uniform pore size and high pore density. However, the lack of biocompatibility is a disadvantage of inorganic membranes.

##### Microcontainer (epoxy-polymer)

Another possibility for macroencapsulation is a newly developed microcontainer composed of a nanoporous epoxy-based polymer [26]. The microcontainer device is produced through the use of adhesion layering techniques and offers several advantages over other macroencapsulation devices, including a high degree of precision associated with the automated manufacturing process, greater durability, small size to prevent clumping, and the ability to determine islet viability in vivo via use of noninvasive procedures such as functional magnetic resonance imaging [26]. There is, however, further testing and research needed in order to optimize the islet loading process, in addition to the need to determine the long-term functionality of the islets.

### Microencapsulation of pancreatic islets

In microencapsulation, one or several islets are encapsulated within a hydrogel, and a number of these microcapsules are used for transplantation. Microencapsulation is used to enclose cells using hydrogels to form a polymer microcapsule ranging in size from zero to several hundred micrometers, and first mentioned by Chang [27]. The benefits of microencapsulation over macroencapsulation include increased surface area, which promotes increased diffusion, which is beneficial for cell oxygenation and glucose stimulated insulin release. However, there are disadvantages to microencapsulation, such as difficultly in retrieval after implantation [11]. Microencapsulation uses various hydrogels composed of alginate, agarose, polyethylene glycol (PEG) etc. to provide an immune barrier for the islets [11].

#### Microencapsulation using alginate

Alginate hydrogel is the most widely used material used for microencapsulation. Alginate microcapsules are made to contain islets through emulsification [28, 29]. Islets are suspended in the alginate/polymer blend; then, calcium ions are added, which force the material to emulsify around the islets [30]. Alginate microcapsules can be fabricated using a microdroplet generator by two-phase aqueous emulsification which can generate a thin alginate hydrogel layer [31]. Optimization of the size of alginate microcapsules is important for islet viability, surgical grafting risk, and metabolic or nutrient supplies. The alginate microcapsule size can be controlled by adjustment of the physical and chemical parameters of the microdroplet generator [31].

When multiple islets are suspended in an alginate capsule, they have been shown to clump together leading to necrosis of the islets in the center of the cluster due to hypoxia. This results in a reduction in the efficacy of the transplant. There is a need to ensure sufficient revascularization and to minimize clumping while maintaining immune protection. Alginate is derived from seaweed and its molecular composition varies depending on the source. This inconsistency may lead to problems with biocompatibility and cytotoxicity. Slight variations in the alginate can lead to different degrees of permeability for insulin, immune cells, and cytokines. There is a large number of groups working on alginate microencapsulation, but the animal studies, and especially the large animal studies, are rarely reproduced. Also, there is significant loss in the number of transplantable microcapsules due to the variability in size [7].

The first case of microencapsulation of islets was in 1980 when the Lim and Sun group encapsulated the islets in microcapsules composed of alginate-polylysine-polyethyleneimine [32]. The results showed reversal of diabetes for 3 weeks in STZ-induced diabetic rats, but the results weren’t sustained due to poor biocompatibility of the material. Later, in 1984, another group used alginate-polylysine-alginate microcapsules to transplant islets in rats; this resulted in diabetes reversal for a year [33]. Further study in using alginate-polylysine-alginate showed that it did not cause a decrease in the insulin release in rat pancreatic islets [34]. In 1992, a study with purified alginate for islet encapsulation in dogs showed median insulin independence for insulin-dependent dogs [35]. Another study by the same group showed that alginate microcapsules with sufficient beta cell mass were able to respond to increased blood glucose concentrations without overshooting hypoglycemia [36]. A recent study showed long-term glycemic correction in rats using triazole-thiomorpholine dioxide alginate microcapsules. Glucose responsive mature beta cells that were derived from human embryonic stem cells were encapsulated in an alginate derivative and transplanted into STZ-induced diabetic rats. The results showed normoglycemia for 174 days without immune suppression, at which point the implant was removed [37].

The first clinical case of human islet transplantation was reported by the Soon Shiong group in 1994 using high guluronic acid alginate microcapsules with human islets [38]. The results showed insulin independence 9 months after the procedure, but the patient was on low-dose immune suppressant drugs. Another group conducted a clinical trial on two non-immune suppressed patients using alginate microcapsules that were double coated with poly-l-ornithine and sodium alginate. The patients showed improvement in their blood glucose levels and a decrease in the daily insulin intake, but insulin independence was not achieved [28]. This study was later repeated in two more patients and showed similar results in 2011 [39]. These improvements were not permanent and the patients reverted to their insulin regimen at the end of the trials.

#### Microencapsulation using agarose

**Fig. 6:**
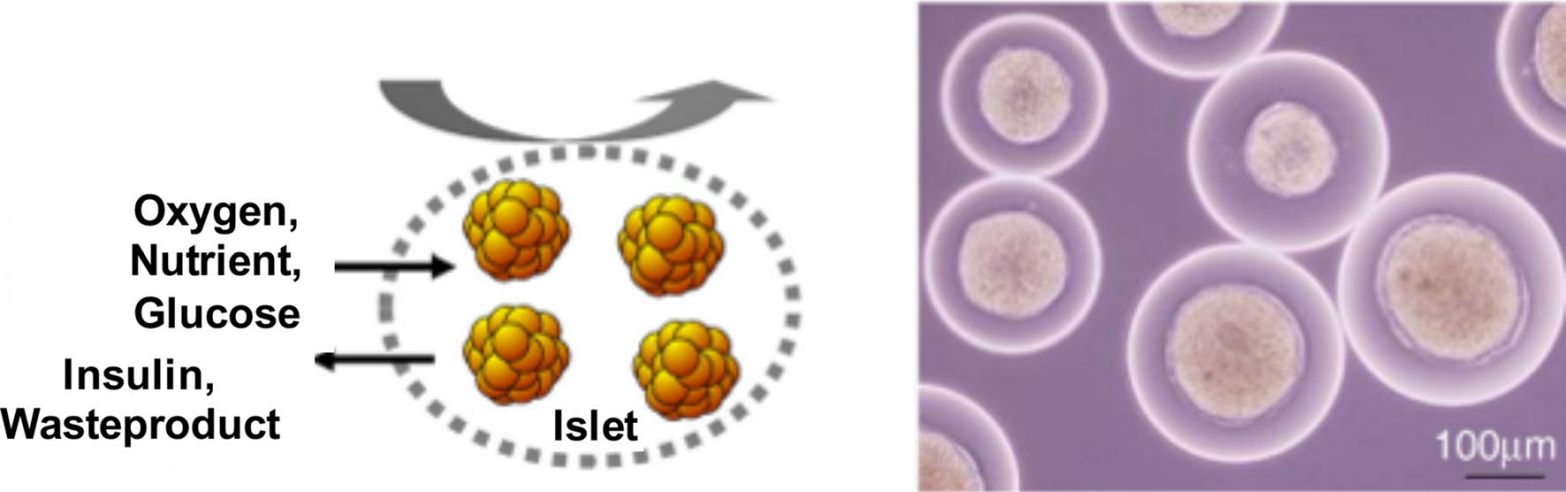
Bioartifical membrane. The concept of immune isolation (*left*). Islet encpasulated in an agarose bead (*right*). Reused with permission from [40]

Another major material that has been used in microencapsulation of islets is agarose. In agarose encapsulation, islets are suspended in an agarose solution which is warmed to 40 °C and agitated to suspend the agarose solution in liquid paraffin. The agarose solution droplets are induced to gel by placing the tubes of solution on ice, encapsulating the islets within agarose beads (Fig. 6) [40]. The effects of the agarose encapsulation on islet survival were tested by implantation into STZ-induced diabetic mice and NOD mice. In the STZ-induced diabetic mice, normoglycemia was achieved for over 100 days. The NOD mice showed normal blood glucose levels for 80–100 days [40]. The potential of immunoisolation was also tested for the prevention of autoimmune recurrence [41]. Islets were isolated from normal healthy mice and encapsulated into the agarose solution and transplanted into NOD mice. The naked islet grafts as a control were destroyed, resulting in recurrence of diabetes in two weeks. However, the encapsulated islets showed normoglycemia for 100 days. The microencapsulated islets showed that the beta cells were well-granulated and not infiltrated by immune cells, indicating that the encapsulation was able to protect the cells from auto-immune destruction [41]. Another research was also done using agarose beads for islet xenotransplantation: hamster islets were transplanted into rats and the results showed normoglycemia for at least 100 days [42]. Based on the above results, agarose microencapsulation can provide complete protection against infiltration by immune cells as shown when implanted into NOD mice. In the future, agarose could have practical applications in humans if it is used to transplant insulin-producing cells made from induced pluripotent stem cells (iPS). In this case, there would be no immune rejection [7].

#### Microencapsulation using polyethylene glycol

Pancreatic islets have also been enclosed in microcapsules via interfacial photopolymerization of PEG-based macromeres. Microcapsules are formed when a dye in the cells is laser-excited, producing free radicals which trigger crosslinking. This crosslinking forms a PEG capsule around the islets. By using different dyes and concentrations and changing the irradiation parameters, the thickness of the capsule can be idealized, typically to around 50 µm. This type of microcapsule is smaller than the others mentioned previously, thus reducing the injection volume. The very thin coatings created in interfacial photopolymerization are not very biodurable due to polymer instability and insufficient crosslinking. Recently, studies have been done researching the effects of crosslinking density and its effects on protein diffusion; increased density delayed protein diffusion through the polymer membrane [43]. The lack of biodurability limits this method’s effectiveness in vivo because the capsule structure may not maintain its integrity. The dyes used in the photopolymerization process also reduced the functionality of the beta cells as insulin secretion decreased significantly with increasing dye concentration [44]. Some studies have shown that PEG crosslinking can result in >90 % islet viability and >90 % encapsulation efficiency. Recent studies have used microfluidics to create PEG capsules that have constant coating thickness instead of constant capsule diameter. This was shown to have the same functionality as normal islets, also preventing loss of function during ex vivo culture [45].

In order to enhance the bioactive properties of the PEG, other peptides such as GLP-1 and ephrinA5-Fc have been immobilized along with PEG, improving the islet viability and functionality [46, 47]. The immobilization of bioactive GLP-1 within PEG hydrogels is efficient and does not alter the bulk hydrogel properties. Further, the GLP-1 immobilized PEG hydrogels enhance the survival and insulin secretion of encapsulated islets. Together with the cell-adhesive peptide RGDS, the immobilized fusion proteins (EphA5-Fc and ephrinA5-Fc) synergistically increased the survival of both MIN6 β-cells and dissociated islet cells, both at a very low cell-packing density (<2 × 10^6^ cells/mL) [46, 47]. Anti-Fas monoclonal antibodies have also been conjugated to the surface of PEG, providing a degree of immunoisolation: coatings containing anti-Fas antibody induced significant T cell apoptosis (21 ± 2 % of cells) after 24 h [48]. However, anti-Fas antibodies only provide protection against T cells and not the other types. In addition, to protect islets from cytokines such as TNF-α, PEG can be functionalized with WP9QY, a peptide which inhibits the negative effects of TNF-α [49]. However, these methods may target a mechanism which is too specific to provide complete immune protection.

#### Microencapsulation using peptide amphiphile

**Fig. 7:**
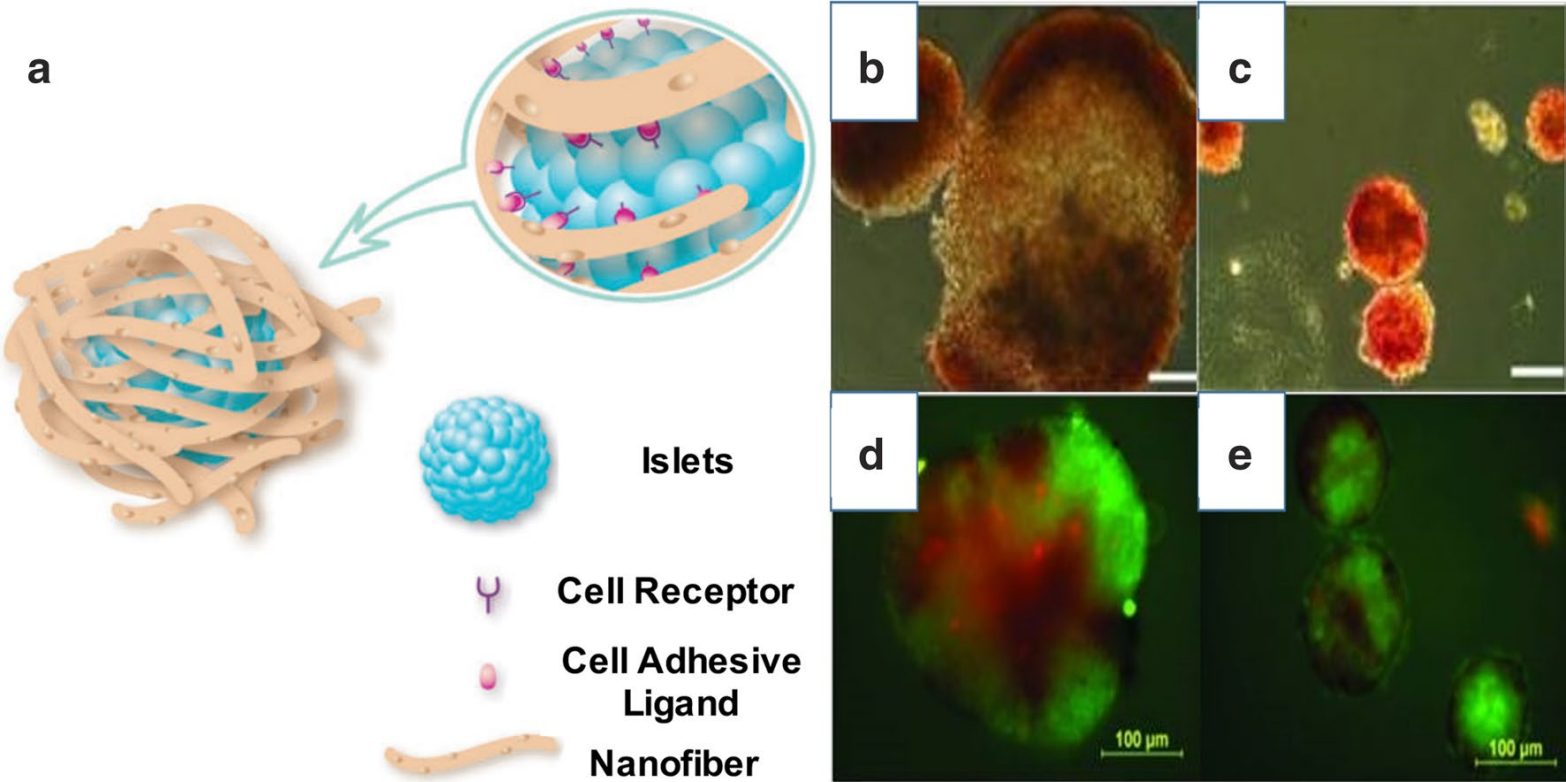
Pancreatic islet encapsulation with PA nanomatrix gel. **a** Scheme of islet encapsulation. **b**–**e** Insulin producing beta cell staining and islet viability test after 14 days of islet culture. Dithizone staining of (**b**) bare islets and (**c**) PA encapsulated islets. Live/dead staining of (**d**) bare islets and (**e**) PA encapsulated islets. In **b** and **c**, *red* shows insulin producing beta cells. In **d** and **e**, live cells are stained *green*, and dead cells are stained *red*. Reused with permission from [50]

Pancreatic islets can be microencapsulated within a nanomatrix gel composed of peptide amphiphiles (PAs). PAs form an extracellular matrix (ECM)-mimicking environment to enhance islet viability without inhibiting functionality while providing an immunoisolation environment. The PA consists of a hydrophilic peptide attached to a hydrophobic C16-carbon chain [50, 51]. The hydrophobic alkyl tails arrange into micelles which form long self-assembled nano scale fibers; then, at the correct pH and following the addition of calcium ions, the self-assembled nanofibers form the nanomatrix gel [50–54]. The peptide sequence can be manipulated to possess various properties of an ECM-mimicking environment such as cell adhesive ligands and enzyme mediated degradation, which enhances the bioactivity and biocompatibility of PAs [55–57]. The PA nanomatrix gel provides an islet-nurturing environment which improves islet viability and function [50, 58]. Furthermore, it provides a semi-permeable barrier which prevents islet exposure to the immunocompetent cells while allowing oxygen, insulin, and nutrient transfer through the nanomatrix. PAs self-assemble without chemical means, which indicates that there is a decreased risk for cytotoxicity stemming from toxic chemicals and dyes in the microcapsule. The chemical makeup of the PAs is carefully controlled during synthesis, resulting in a low probability of potentially harmful material variation. Several studies have shown the beneficial effects of PA nanomatrix gel encapsulation on islet viability and function. One study was conducted in which rodent islets were incorporated into the PA nanomatrix gel containing a cell-adhesive ligand, arginine-glycine-aspartic acid (RGD), as well as a MMP-2 sensitive sequence [50]. The PA-RGD nanomatrix gel-encapsulated islets maintained insulin secretion function and islet viability over 14 days, while the unencapsulated islets showed a marked decrease in both function and viability (Fig. 7) [50]. In addition, another group developed a heparin-binding PA nanomatrix gel, which enhanced angiogenic activity of the nanomatrix gel [58]. Delivery of FGF-2 and VEGF along with the heparin-binding PA nanomatrix gel demonstrated improved islet viability and function along with enhanced angiogenesis. For in vivo application of a PA nanomatrix gel, a bio-inspired hybrid nanosack was developed by combining a PA nanomatrix gel and an electrospun poly (ε-caprolactone) (ePCL) nanofiber sheet with porous crater-like structures [59]. The PA nanomatrix gel provides an islet-nurturing environment, while the ePCL nanofiber sheet maintains the mechanical stability of the gel within the implant area. In addition, the delivery of FGF-2 along with the crater-like structures of the ePCL nanofiber sheet synergistically benefited blood vessel formation in the hybrid nanosack when implanted into the rat omentum. Thus, the hybrid nanosack shows potential to be used as a bioartifical pancreas since it provides an islet-protective and nurturing environment along with enhanced angiogenesis in the implantation area [59].

### Surface modification of pancreatic islets

Surface modification is a form of immunoisolation that does not rely on microencapsulating islets, but rather on altering the surface of the islets themselves to form an immune barrier. A PEG complex may be covalently bound to the surface of the islets to provide a thin barrier to macrophages and reduce the release of cytokines. For example, a succinimidyl ester-functionalized PEG can react with amine groups present upon the cell surface of pancreatic islets to conceal host immunogenic surface antigens [60]. PEG was shown to very effectively block the effect of splenocytes, a type of macrophage. However, islets coated with PEG were shown to still be vulnerable to harmful cytokines like TNF-α [61]. This raises a significant concern regarding surface modification. In fact, direct covalent surface modification can affect normal cellular functions which are associated with cell surface molecules [62]. It may be difficult to fully immunoisolate the islets from all of the macrophages and cytotoxic cytokines with such specific surface molecules. The strong covalent bond utilized in conjugation may also alter the physiology of the islet clusters. The insulin output of modified islets was shown to be different over time compared with unmodified islets. The functionality of PEG surface-treated islets decreased even though most of the cells were still shown to be viable. After a longer period of surface modification, PEG may infiltrate and interact with the islets, causing necrosis [63].

As an alternative approach, an electrostatic adsorption has been proposed, in which poly(l-lysine)-graft-poly(ethylene glycol) (PLL-g-PEG) copolymers were physically coated onto islet interfaces [64]. Although this approach can attain noncovalent surface modification of pancreatic islets, the inherent cytotoxicity of PLL polymers hampers the safety of this approach. To create a functional coating for improving response to glucose, a layer-by-layer (LbL) self-assembly technique has been studied. Wilson et al. formed nano-thin conformal coatings on individual pancreatic islets using LbL self-assembly of poly (l-lysine)-g-poly(ethylene glycol)(biotin) and streptavidin (SA), and showed comparable functionality of the LbL-modified islets compared to non-coated controls in a murine model of allogeneic intraportal islet transplantation [65].

### Bioartificial pancreas applications with microfluidic or micropatterning technology

According to the previous summary about immune protection for implanted islets, several types of biomaterials and devices were fabricated using microfluidic or micropatterning technology. On the basis of the technical support, both microfluidic devices and micropatterned surfaces have been introduced to generate precise micro-scaled encapsulation in order to transfer islets and to protect islets against the body’s immune responses. For example, particles [45] and fibers [66, 67] for islet microencapsulation were produced by the multiplexed droplet generating technique with microfluidic devices based on an oil–water interface. Moreover, islets were also encapsulated in an air–water interface without using harmful immersion oil [68], using a hydrogel capsule as an effective nano- or micro-scaled protection layer against inflammatory responses [69]. While generating hydrogel particles or fibers for islets has advantages in a multiplexed and rapid manufacturing process, they still have challenges for assembly into massive constructs to transplant. The total number of viable islets is one of the most important factors to recover the normal level of insulin secretion. However, islets can often be damaged during the fabrication process in a microfluidic device due to the high shear stress condition required for the droplet generation. Although the multiplexed generation of droplets can provide a large number of products at once, it is challenging to maintain high cellular viability. On the other hand, a micropatterning technique has provided a simple, precise approach for constructing a massive hydrogel structure at once, and for generating multicellular clusters as islet-like cellular aggregates using single distributed islets or beta cells in highly packed array patterns [70–72]. Moreover, these kinds of approaches can also provide technically improved transplantable islets using not only restricted islets, but also differentiated stem cells or different types of cells for co-culture, which can be alternative sources of islets for transplantation.

The development of microfluidic devices has largely switched to the multifunctional monitoring system for isolated islets [73–78]. The biological function of a single islet inside a microfluidic device has been examined for intracellular Ca^2+^ [73, 75], amino acids [78], cellular impedance [77], or insulin secretion [74, 76] under dynamic external stimuli. Various designs of microfluidic devices enable the capture of an isolated islet in a microfluidic channel and the analysis of their biofunctional metabolites, e.g. insulin secretion, Ca^2+^ influx, and apoptotic factors based on the chemical gradient or dynamic culture conditions. Although technical improvement has been supported for real-time, accurate, and multifunctional investigational methods using even a single islet, there is still a great need for an intensive microfluidic device to assess the immune response from bare or encapsulated islets and to assist in pretreatment of islets before transplantation. Although many groups have attempted to encapsulate islets for protection against inflammatory responses, producing the outer structure as a carrier, the inner structure of the islets has been overlooked. For instance, flow-induced culture conditions were applied to islets by Sankar et al. [79] and demonstrated the importance of microvascularization inside the isolated islets. Without the microsvaculature within the islets, it is difficult to maintain or sustain isolated islets for more than a few weeks. It is critical not only for intercellular restoration of restricted diffusion in vitro, but also for longer maintenance of ex vivo islets within flow-induced culture conditions. The encapsulated islets could be exposed to physiological flow conditions as a pretreatment process in order to stabilize the islets properly before transplant.

## Conclusions

A bioartificial pancreas is a therapeutic approach to enable immunoisolation of transplanted islets, which allows allo- and xenotransplantation of islets without or with minimal immune suppression. In this review, we have discussed various types of bioartificial pancreases including macro- and microencapsulation and islet surface modification; each strategy has some advantages and limitations. Macroencapsulation approach is useful for enclosing islets with relative ease of retrieval if necessary. However, there is a concern for clumping of the islets, which could potentially reduce oxygen and nutrient flow to the cells on the interior of the cluster. Microencapsulation approach is recently the most widely utilized strategy to encapsulate single or several islets, because it is favorable for substance exchange due to its large surface area. However, microencapsulated islets are hardly retrievable once implanted into the body, and the size or thickness of the gel and the permeability should be optimized for this application. Surface modification approach enables the generation of a very thin and finely-controlled immune barrier coating against the host immune system, but it may alter the physiology of the islets during the modification process and the coating stability should also be improved for long-term islet transplantation. From a technical point of view, microfluidic or micropatterning technology enables the development of various tools in bioartificial pancreas application, such as rapid, precise, multiplexed fabrication of nano- and micro-sized encapsulation of transplantable islets using various materials and compositions. Although previous technical development of microfluidic devices has been more focused on in vitro monitoring systems, they may also have great technical potential for encapsulating and transferring islets in the bioartificial pancreas.

In interest of future applications of pancreatic islet transplantation, recent advances in the developmental biology of pancreatic organogenesis have enabled researchers to attempt the generation of fully differentiated pancreatic beta cells from embryonic stem cells or iPSCs; a bioartificial pancreas may also provide great potential for the transplantation of stem cell-derived beta cells. Despite much progress in the development of the bioartificial pancreas, its clinical applications are few and the clinical outcomes are not clear. Therefore, much effort is still needed to overcome the limitations of bioartificial pancreas applications.

## Abbreviations

RGD: arginine–glycine–aspartic acid
ePCL: electrospun poly (ε-caprolactone)
ECM: extracellular matrix
iPS: induced pluripotent stem cells
IVGTT: intravenous glucose tolerance tests
LbL: layer-by-layer
NOD: non-obese diabetic
PAs: peptide amphiphiles
PEG: polyethylene glycol
PLL-g-PEG: poly(l-lysine)-graft-poly(ethylene glycol)
PTFE: polytetrafluoroethylene
SA: streptavidin
STZ: streptozotocin
